# Hybrid deep learning-based multimodal framework for plant leaf disease classification using RGB, Excess Green (ExG), and pseudo-thermal representations with MobileNetV2

**DOI:** 10.1038/s41598-026-52115-4

**Published:** 2026-05-21

**Authors:** Saba Begum, E. Naresh, N. N. Srinidhi

**Affiliations:** 1 Manipal Institute of Technology Bengaluru, Manipal Academy of Higher Education, Manipal, India; 2 Department of Computer Science and Engineering, B.M.S. College of Engineering, Bengaluru, Karnataka India

**Keywords:** Plant disease detection, Multimodal learning, MobileNetV2, Excess Green (ExG) vegetation index, Pseudo-thermal representations, Ginger Leaf Dataset, Computational biology and bioinformatics, Mathematics and computing, Plant sciences

## Abstract

Plant diseases are a serious danger to the world’s food security, because they lower agricultural output and increase economic losses. Due to subjectivity, fluctuating lighting, and environmental unpredictability, traditional visual examination techniques are frequently incorrect. The Excess Green (ExG) vegetation index and pseudo-thermal representations produced from RGB pictures are two synthetically developed complementary representations that are integrated with RGB imagery in this study’s lightweight multimodal deep learning system to address these issues. Histogram shifting and pseudo-infrared color mapping are used in a reproducible picture alteration pipeline to create the pseudo-thermal modality, which allows for extra visual signals without the need for specific thermal sensors. In order to classify plant diseases while preserving computational efficiency, the suggested framework uses MobileNetV3-Small backbones to extract modality-specific characteristics. This is followed by feature-level fusion. The publicly accessible Ginger Leaf Dataset, which includes RGB pictures of ginger leaves in four different conditions—Damage-Pest, Dehydrated, Healthy, and Leaf-blight—was used for the experiments. For training, validation, and testing, the dataset was split using a stratified 70:15:15 split. Python-based preprocessing procedures were used to create the extra modalities (ExG and pseudo-thermal representations) from the original RGB images. The experimental results show that the combination of the representations with RGB images can enhance the classification performance compared with the unimodal RGB-based models. Ablation experiments are also conducted to examine the contributions of different modalities to the overall categorization accuracy. The experimental results show that plant disease recognition can be improved with the help of efficient computing by combining lightweight convolutional neural networks with computationally generated visual representations.

## Introduction

Agriculture is still an important factor in global food and economic security, especially in areas where crops are used as the primary source of income. However, diseases have been a major problem in reducing agricultural production since they lower the yield, quality, and cost of the crops. Therefore, it is crucial to identify diseases that affect the leaves of the plants to avoid damage, improve the application of pesticides, and encourage green practices.

To identify plant diseases, agricultural specialists have long used eye inspection methods. These manual methods are frequently labor-intensive and challenging to apply on a broader scale. But when the experiment is controlled, they work well. These limitations have led to the development of automated techniques for diagnosing plant diseases based on computer vision and machine learning. Convolutional Neural Networks (CNNs) in particular have shown a strong ability to extract discriminative visual information from plant leaf photos in recent years. Several CNN-based models have demonstrated classification accuracy of above 95%^1–3^ under controlled experimental settings. These models are able to identify complex visual patterns, such as lesion shapes, discoloration, and textural variations associated with plant diseases.

Several deep learning models have been investigated in the past to increase the efficiency of plant disease detection. To address problems like feature redundancy and class imbalance in plant disease datasets, for instance, generative models like DC-GAN combined with MDFC-ResNet have been proposed. Using hybrid CNN architectures that combine DenseNet and Inception networks to improve feature extraction skills is another illustration. Despite its potential, RGB-based plant disease detection systems frequently encounter practical challenges because of differences in illumination, overlapping disease symptoms, and a lack of diversity in datasets.

To increase robustness while preserving computing economy, researchers have been looking more and more into hybrid learning techniques and lightweight CNN architectures. For situations with limited resources, models based on MobileNet, ResNet, and SqueezeNet have shown competitive classification performance with much lower computing requirements^3–5^. To improve feature representation and classification accuracy, further research has investigated transfer learning, attention mechanisms, and hybrid CNN–RNN architectures^6,7^.

The addition of other image representation types, which are supplementary to the usual RGB image representation, is another interesting option. It has been suggested that RGB images do not provide an accurate representation of certain vegetative stresses that are related to health issues, though they do provide an accurate representation of certain disease symptoms that are visible on the leaves of plants. In the analysis of vegetation, images derived using the Excess Green (ExG) index are commonly used to identify plant patches and to focus attention on certain changes that are related to chlorophyll. In the same way, other image representations of plant leaves, which are derived using image alteration techniques, are possible.

In order to improve crop monitoring tasks, agricultural computer vision research has also emphasized the application of multimodal and multispectral fusion techniques in recent times. For example, the application of cross-modal fusion techniques, where RGB and near-infrared images are used, has shown promise for improving agricultural monitoring and mapping of vegetation. With regard to field weed mapping, Fan et al^8^. proposed a cross-modal feature fusion framework that uses RGB and near-infrared images. Similarly, Ulku et al^9^. also proposed a cross-band correlation-aware fusion technique for multispectral image analysis.

Inspired by the above-mentioned results, this paper explores the possibility of using a lightweight multimodal approach to plant disease categorization, which uses RGB images in addition to two different representations of RGB images, namely pseudo-thermal representation and the Excess Green (ExG) vegetation index. This paper uses a reproducible image processing pipeline to generate pseudo-thermal images. It is essential to remember that the pseudo-thermal images used in this work are not captured by any camera, but they are derived images.Without the need for specialist imaging equipment, these extra visual representations offer supplemental clues that could enhance disease classification.

The suggested approach uses lightweight convolutional neural network backbones based on MobileNet architectures to extract modality-specific characteristics from RGB, ExG, and pseudo-thermal pictures. A fully linked classification layer receives the extracted features after they have been merged via feature-level fusion. This paper focuses on examining how complementary representations formed from RGB images can improve plant disease recognition within an effective multimodal learning framework, rather than suggesting a totally new fusion process.

Experiments in this study were conducted using the publicly available Ginger Leaf Dataset^10^. The dataset contains RGB images of ginger leaves representing four plant conditions: *Damage-Pest*, *Dehydrated*, *Healthy*, and *Leaf-blight*. Additional ExG and pseudo-thermal representations were generated from the RGB images using Python-based preprocessing pipelines. The resulting dataset was divided into training, validation, and testing subsets using a stratified 70:15:15 split.

### Primary contributions

The primary contributions of this research are summarized as follows:

- Development of a lightweight multimodal plant disease classification framework that incorporates RGB images and two derived representations, namely the Excess Green vegetation index and pseudo-thermal images generated from RGB data.
- Exploration of lightweight MobileNet-based convolutional neural network architectures for effective feature extraction from multimodal plant leaf images.
- Design of a feature-level fusion strategy that integrates complementary visual representations derived from RGB imagery.
- Comprehensive experimental evaluation including modality ablation, fusion analysis, and comparison with alternative architectures such as multi-branch and multi-channel models.

**Fig. 1:**
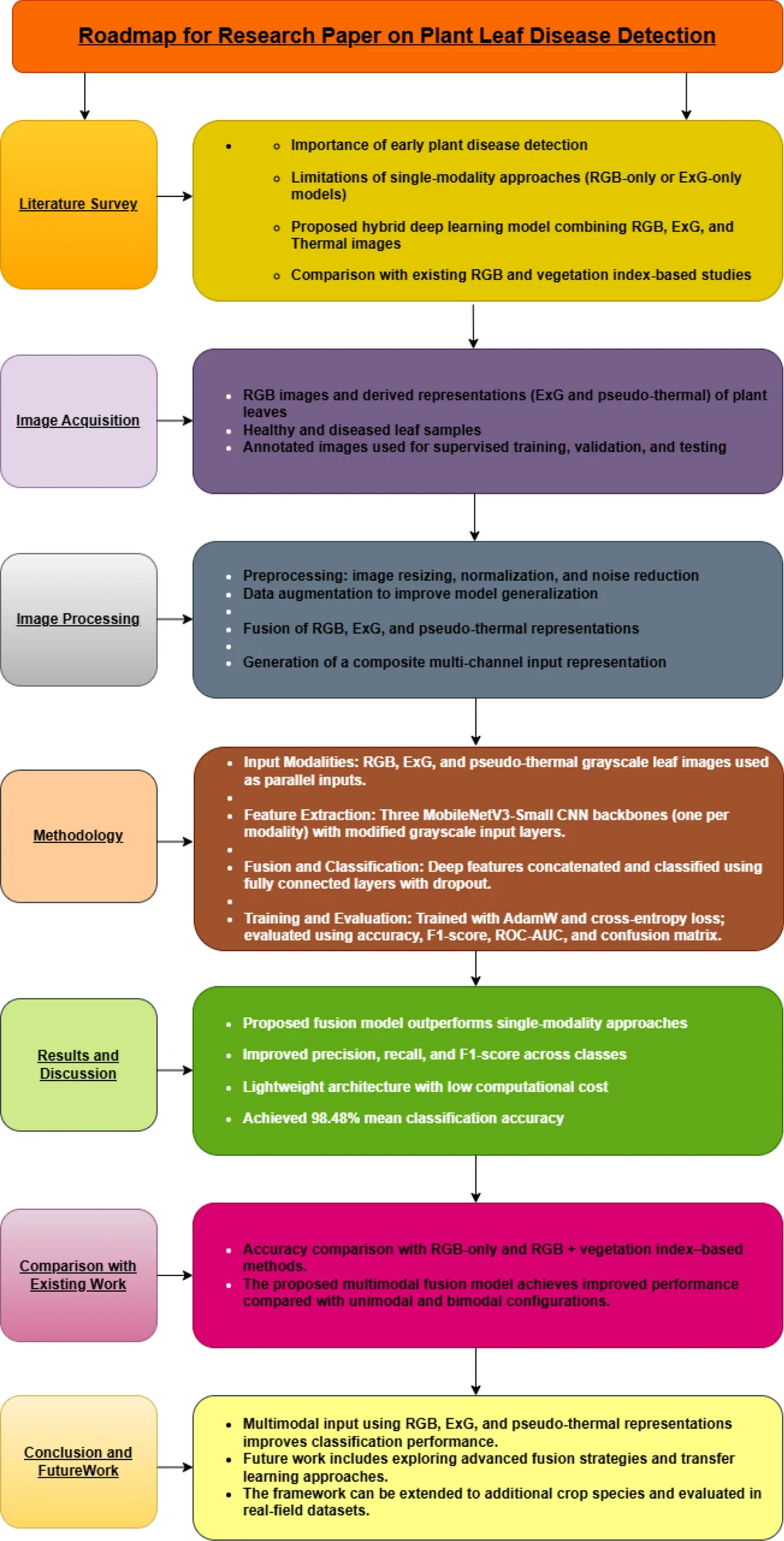
Structured workflow for multimodal plant disease detection using deep learning.

Figure 1 illustrates the overall workflow of the proposed multimodal plant disease detection pipeline. The framework includes stages such as literature analysis, dataset preparation, image preprocessing, generation of ExG and pseudo-thermal representations, multimodal feature extraction using lightweight CNN models, feature fusion, and classification.

**Fig. 2:**
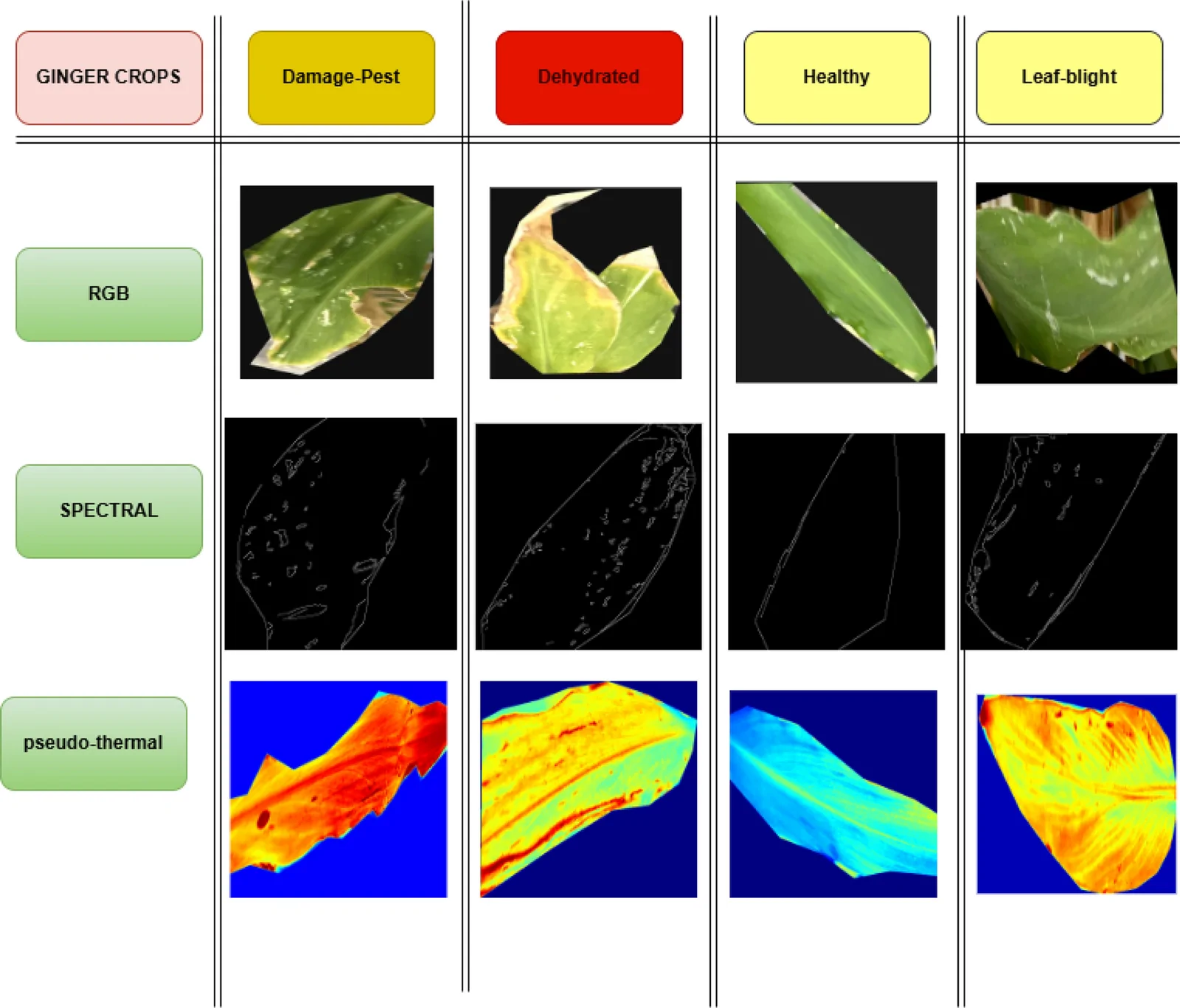
Visual dataset samples from the Ginger Leaf Dataset showing healthy and diseased ginger leaves used for training and evaluation.

Figure 2 presents representative samples from the Ginger Leaf Dataset used in this study. The dataset includes both healthy and diseased ginger leaf images corresponding to four classes: Damage-Pest, Dehydrated, Healthy, and Leaf-blight.

## Related work

Automated plant disease diagnosis has been greatly enhanced by recent advances in deep learning, allowing for scalable and precise detection systems across many crops and imaging modalities. Traditional convolutional neural networks (CNNs), hybrid architectures, transformer-based frameworks, and multimodal sensing systems are just a few of the methods that researchers have investigated. These methods seek to decrease computer complexity for real-world agricultural applications, increase model generalization, and improve disease detection accuracy.

1. Variants of Convolutional Neural Networks (CNNs) CNN-based architectures remain among the most widely used approaches for plant disease detection because of their strong capability to learn hierarchical spatial features from leaf images. For instance, bacterial leaf blight in rice leaf thermal images has been detected with 97.5% accuracy using modified CNN architectures^11^. In a similar vein, popular architectures like VGG16, ResNet18, and MobileNetV2 have proven to perform well in multi-crop plant disease classification tasks^3,4,12^. Lightweight architectures like SqueezeNet^7^, MobileNet^13^, and MobileNetV3^14^ have been created to allow deployment in contexts with limited resources. These models retain competitive accuracy while drastically reducing computing complexity. However, when disease symptoms are visually similar or overlap across classes, lightweight CNN models may find it difficult to capture complicated contextual interactions^4,13,15,16^.
2. Ensemble and Hybrid Architectures There has also been much research done on hybrid architectures that combine traditional machine learning methods with deep learning models. One noteworthy example is the DC-GAN with MDFC-ResNet architecture, which obtained 95.99% accuracy in rice illness classification by addressing feature redundancy and class imbalance by synthetic data augmentation^1^. Similarly, in early-stage rice disease detection tasks, hybrid pipelines combining VGG-16, Faster R-CNN, and Random Forest classifiers obtained 97.3% accuracy^2^. To enhance feature representation, several hybrid frameworks have been developed, such as DenseNet-Inception-Attention networks^17^, CNN-BiLSTM architectures^18^, and Inception-ResNet models optimized using Firefly methods^19^. Although ensemble models such as ResNet101 combined with Random Forest classifiers provide improved interpretability and robustness^20^, their computational cost often limits real-time deployment in agricultural monitoring systems.
3. Attention Mechanisms and Vision Transformers Vision Transformers (ViTs) and attention mechanisms have been investigated recently for the classification of plant diseases. These models are able to extract global contextual information and long-range dependencies from photos. Transformer-based architectures and attention-guided CNN models have been shown to be successful in identifying plant diseases^21–24^. Attention-based networks have been effectively used to improve classification accuracy and localize unhealthy areas in crops like bananas, tomatoes, and maize^17,24,25^. However, compared to lightweight CNN models, transformer-based architectures usually need more processing power, which may limit their applicability for deployment on edge devices in agricultural monitoring systems.
4. Sensor-Based and Multimodal Systems Numerous studies have looked into merging different sensory modalities beyond traditional RGB imagery to increase robustness and diagnostic accuracy. In agricultural applications, multispectral imaging systems that combine thermal, hyperspectral, and near-infrared (NIR) data have shown enhanced disease detection capabilities^21,26,27^. For instance, rice illnesses have been identified using RGB and thermal image fusion in a variety of environmental settings^28^. However, these systems usually rely on sophisticated acquisition configurations and specialized sensors, which raise the infrastructure and implementation costs. In order to improve vegetation analysis, cross-modal fusion approaches that incorporate complementary spectral information have also been studied recently. Fan et al.^8^ have shown that incorporating complementary spectral information enhances vegetation classification performance by proposing a cross-modal feature fusion framework for field weed mapping using RGB and near-infrared data. In a similar vein, Ulku et al.^9^ emphasized the significance of modeling correlations between spectral bands to improve feature representation when introducing a cross-band correlation-aware fusion strategy for multispectral image analysis. These experiments demonstrate how multimodal fusion techniques work well for agricultural vision tasks. Some studies have investigated the creation of complementary image representations from RGB images using image processing techniques, in contrast to sensor-based multimodal systems. Although they may not represent physically measured modalities, such representations can offer extra visual cues without the need for specialized sensors.
5. Explainability, Optimization, and Model Interpretability Agricultural AI systems now need to be interpretable. Agronomists have made extensive use of explainable AI approaches as Grad-CAM, SHAP, and LIME to enhance transparency and display model predictions^23,29,30^. Additionally, optimization methods have been used to enhance model convergence and performance. Techniques like Firefly Optimization^19^, Whale Optimization Algorithm^31^, and Adaptive Deep CNN-RNN models (ADCRNN)^7^ have shown increases in classification accuracy. Nevertheless, many of these models have little validation in actual agricultural settings and are mostly assessed in restricted experimental settings.
6. Edge Computing, IoT Integration, and Lightweight Deployment Several studies have looked into implementing lightweight plant disease detection algorithms on edge computing systems to support real-time agricultural monitoring. Embedded systems such as Raspberry Pi and NVIDIA Jetson devices have been used to create lightweight CNN models such multiscale CNNs^32^, quantized ResNet architectures^12^, and MobileViT networks^13^. There has also been research on IoT-enabled smart agriculture systems that incorporate sensors, data analytics, and automated decision support mechanisms^13,33,34^. Real-time crop monitoring and automated agriculture management procedures like pest and irrigation control are made possible by these technologies^25,35^. Despite these developments, model resilience in real-world agricultural settings is still impacted by issues like environmental unpredictability, dataset imbalance, and a lack of field datasets.
7. Emerging Methods and Sustainable Disease Management In addition to AI-based detection technologies, recent studies have looked into ecologically friendly methods of managing plant diseases. In a number of crops, nanotechnology-based therapies like silver nanoparticles (AgNPs) and metal nanoparticles containing copper and zinc have shown potent antifungal and antibacterial qualities^36,37^. Biological control agents including bacteriocins, bacteriophages, and fungal biocontrol organisms such as *Purpureocillium lilacinum* have also been investigated for environmentally friendly pathogen suppression^38,39^. In addition, chitosan and nano-chitosan treatments have shown potential for enhancing plant immunity while reducing reliance on chemical fungicides^40^.
8. Multidisciplinary and Emerging Research Directions The integration of artificial intelligence, embedded systems, and sustainable agricultural technology is highlighted in recent multidisciplinary studies published in journals like *Computers and Electronics in Agriculture*. Fuzzy logic-based IoT-based pest monitoring systems have shown promise for real-time crop monitoring while lowering pesticide use^37^. For high-performance agricultural image analysis, hybrid deep learning architectures that combine CNNs and Vision Transformers are being investigated more and more^41,42^. For instance, Borhani et al.^42^ suggested a hybrid ViT-CNN architecture that balances computational efficiency and predictive performance for edge computing contexts. Surveys also highlight the importance of using consistent datasets and validation across different crops and using unified evaluation criteria^43,44^.

The main shortcomings found in current plant disease detection systems are compiled in Table 1. These drawbacks include restricted access to a variety of annotated datasets, poor cross-crop generalization, difficulties implementing lightweight models in actual agricultural settings, and a lack of established benchmarks. Furthermore, a lot of multimodal techniques depend on specialized sensing equipment, which raises the cost and complexity of the system.

**Table 1:** Critical research gaps in deep learning-based plant disease detection.

**Research gap**	**Description and supporting studies**
Multi-crop and multi-disease generalization	Most models perform well on specific crops or diseases but often fail under cross-domain scenarios or multi-disease settings, lacking adaptability and robustness across field conditions ^1,11,44^
Lack of unified benchmarks	There is a lack of standardized evaluation protocols and benchmark datasets, making it difficult to compare models fairly across studies ^22,43^
Resource-constrained deployment	Lightweight architectures designed for edge devices may still struggle in uncontrolled field conditions due to noise, illumination variation, and hardware constraints ^13,15,44^
Dataset deficiency	High-quality annotated field datasets are limited, restricting model generalization and validation across seasons and geographic regions ^22,29,44^
Explainability and trustworthiness	Few models integrate interpretability directly into their architecture, which reduces agronomist trust and practical deployment potential ^23,30,42^
Limited multimodal accessibility	Many multimodal approaches rely on specialized sensors such as hyperspectral or thermal cameras, which increases system cost and limits scalability for practical agricultural deployment ^8,9,28^

**Table 2:** Comparison of representative plant disease classification approaches with the proposed framework.

**Sl. No.**	**Study (Abbrev.)**	**Model/technique**	**Modality**	**Accuracy**	**Key characteristics**
1	Hybrid rice detection	DC-GAN + MDFC–ResNet	RGB	95.99%	GAN-based data augmentation with deep feature extraction
2	Thermal-aware prediction	Modified DCNN	Thermal imagery	97.5% (Precision)	Thermal-based crop stress detection
3	Multi-model fusion	VGG-16 + R-CNN + RF	RGB	97.3%	Hybrid architecture with feature-level fusion
4	Dense-inception classifier	DenseNet + Inception + Attention	RGB	Not reported	Multi-scale contextual feature extraction
5	Lightweight squeezenet	SqueezeNet + SMOTE	RGB	97%	Lightweight model with class balancing
6	Proposed model	MobileNetV3 fusion framework	RGB + ExG + Pseudo-Thermal	98.48%	Lightweight multimodal fusion using derived representations

**Table 3:** Comparison of input modalities and sensors.

**Model**	**Modality**	**Sensors used**	**Spectral bands**	**Application domain**
DC-GAN + MDFC-ResNet	RGB	Standard RGB camera	400–700 nm	Rice disease detection
Modified DCNN	Thermal	FLIR thermal camera	7.5–13.5${\upmu }$m	Stress monitoring
VGG + R-CNN + RF	RGB	DSLR/Phone camera	400–700 nm	Early detection
DenseNet + Attention	RGB	RGB camera	Visible spectrum	Crop classification
SqueezeNet + SMOTE	RGB	RGB camera	Visible spectrum	Maize disease detection
Proposed (Ours)	RGB + ExG + Pseudo-Thermal	RGB camera + index generator	Visible + derived representations	Multicrop disease classification

A comparative summary of exemplary deep learning models for plant disease detection is shown in Table 5, which highlights the models’ architectures, input modalities, reported performance, and main benefits. The comparison shows that RGB imagery or computationally demanding hybrid designs are the mainstays of many current methods. On the other hand, in order to improve classification performance while preserving computational economy, the suggested framework investigates the integration of complimentary picture representations produced from RGB images (Table 2).

The input modalities and sensing requirements utilized by various plant disease detection models are compared in Table 3. While multispectral systems necessitate additional hardware infrastructure, the majority of current methods depend on conventional RGB cameras or specialized heat sensors. By using image processing techniques to instantly create complimentary ExG and pseudo-thermal representations from RGB images, the proposed framework, on the other hand, does not require specialized imaging sensors.

## Materials and methods

This section describes the dataset used in this study, the generation of derived image representations, preprocessing procedures, the multimodal network architecture, the feature fusion mechanism, the training configuration, and the evaluation protocol.

### Dataset description

This study utilizes the publicly available Ginger Leaf Dataset^10^, which contains RGB images of ginger leaves collected under natural field conditions. The dataset includes images representing multiple ginger leaf health conditions including *Damage-Pest*, *Dehydrated*, *Healthy*, and *Leaf-blight*.

All images in the dataset are originally captured as RGB images. In this work, additional derived representations were generated from the RGB images, including simulated Excess Green (ExG) vegetation index and pseudo-thermal representations. These derived modalities are computational transformations of RGB imagery rather than measurements obtained from independent sensors.

To ensure reproducibility and balanced class distribution, the dataset was divided using a stratified **70:15:15** split for training, validation, and testing.

**Table 4:** Dataset split used for model training, validation, and testing.

**Dataset split**	**Number of samples**	**Percentage**
Training set	7635	70%
Validation set	1634	15%
Test set	1641	15%
Total	10910	100%

The dataset composition and distribution across splits are summarized in Table 4. Each RGB image was paired with two derived representations: an Excess Green (ExG) index image and a pseudo-thermal representation generated from the RGB image.

### Derived image representations

Since the Ginger Leaf Dataset dataset provides only RGB images, additional representations were generated using image processing techniques to provide complementary visual cues. These representations are derived from RGB imagery and do not correspond to measurements from physical sensors.

#### Excess Green (ExG) vegetation index

The Excess Green index emphasizes vegetation intensity and highlights variations in chlorophyll content. The ExG index is computed using Equation 1.

1 $$\begin{aligned} ExG = 2G - R - B \end{aligned}$$

where *R*, *G*, and *B* represent the red, green, and blue channel intensities. The computed values are normalized to the range *[0,1]* and stored as single-channel grayscale images.

#### Pseudo-thermal image generation

A pseudo-thermal representation was generated from RGB images using a deterministic image processing pipeline. The procedure includes:

1. Conversion of RGB images to grayscale
2. Histogram intensity shifting
3. Application of a pseudo-infrared colormap

These transformations produce temperature-like visual gradients that may highlight structural patterns in leaf textures, although they do not represent real thermal sensor measurements.

### Image preprocessing and data augmentation

All images were resized to *224 × 224* pixels to match the input requirements of the network architecture. RGB images were normalized using ImageNet statistics, while ExG and pseudo-thermal representations were normalized using a mean of *0.5* and a standard deviation of *0.5*.

To improve model robustness and generalization, the following augmentation techniques were applied during training:

- Random horizontal flipping
- Random affine transformations
- Color jittering
- Brightness and contrast adjustments

**Fig. 3:**
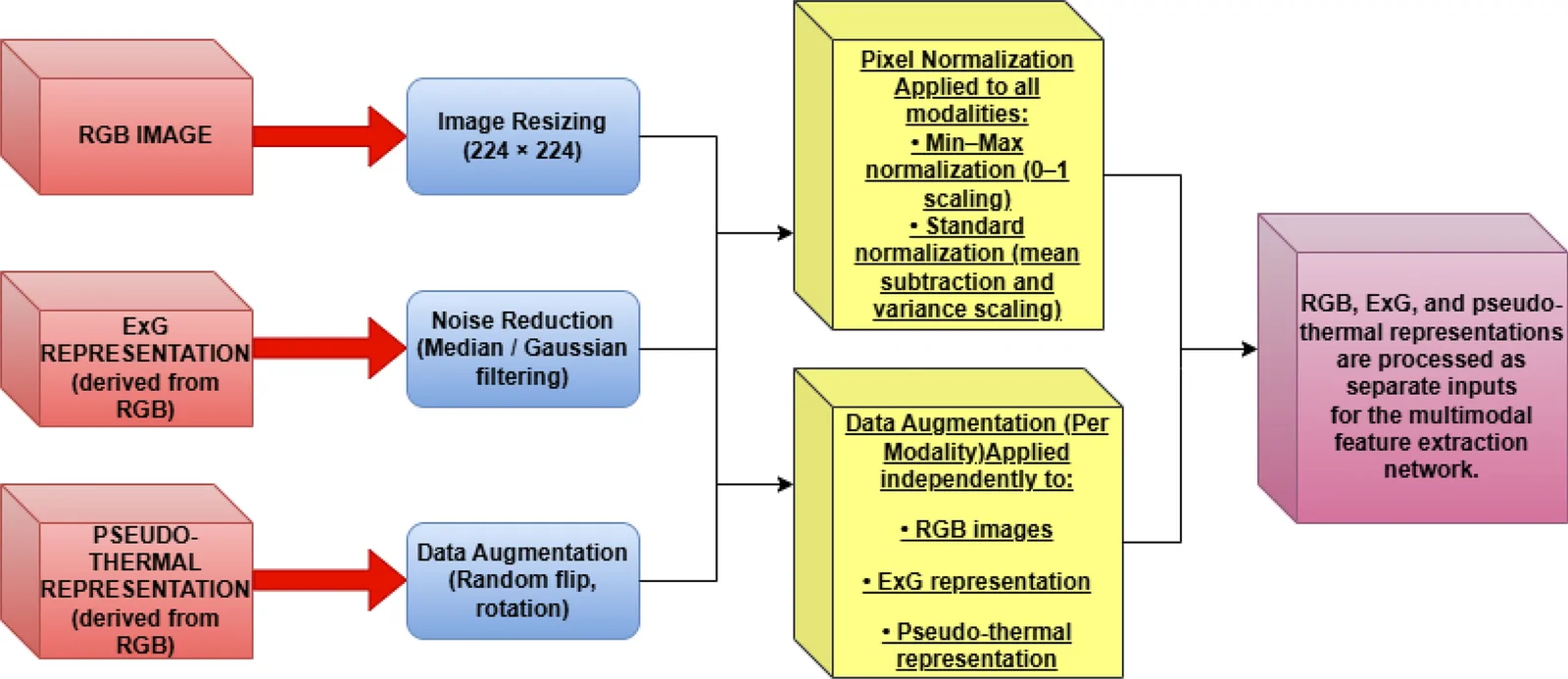
Preprocessing pipeline used to generate RGB, ExG, and pseudo-thermal representations.

The preprocessing workflow for generating the multimodal inputs is illustrated in Figure 3.

### Multimodal network architecture

The proposed framework employs a multi-branch convolutional architecture in which each modality is processed by a separate encoder. Three modality-specific encoders are used:

- RGB encoder
- ExG encoder
- pseudo-thermal encoder

Each encoder uses a MobileNetV3-Small backbone initialized with ImageNet pretrained weights. The final classification layer of the backbone is removed to obtain feature embeddings.

For single-channel modalities (ExG and pseudo-thermal), the first convolution layer is modified to accept one input channel.

Each encoder produces a feature vector of dimension:

*d = 576*

**Figure 4:**
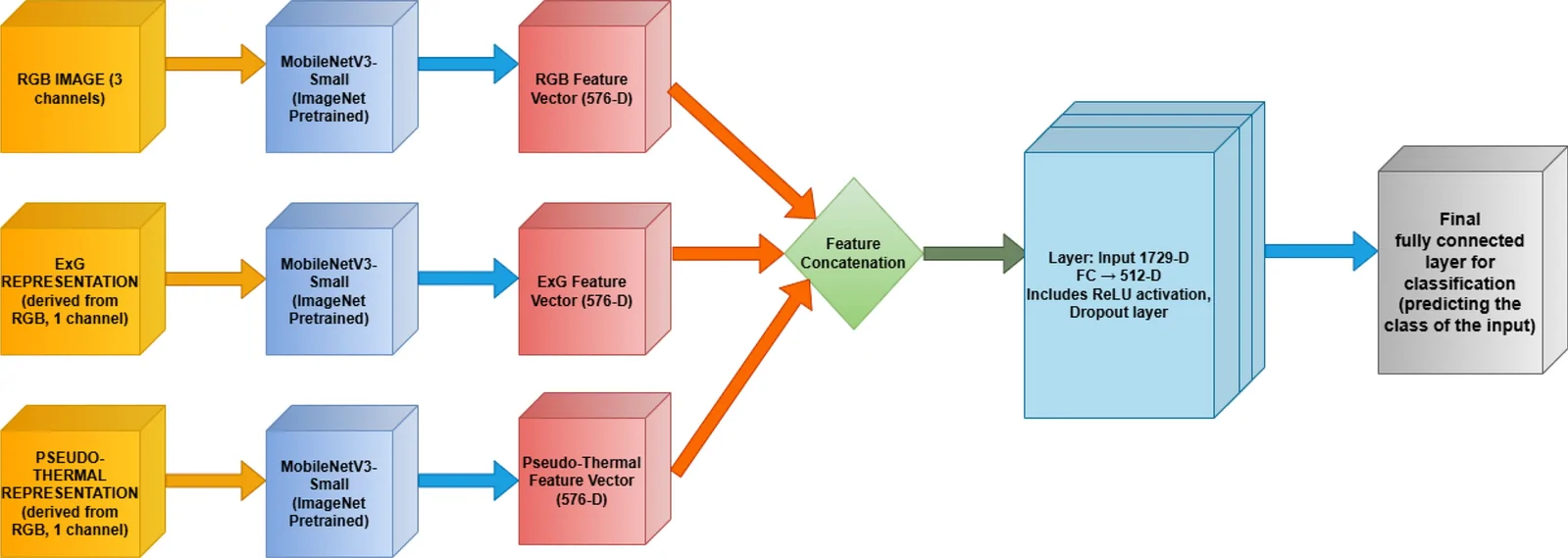
Multimodal feature extraction and fusion pipeline.

**Figure 5:**
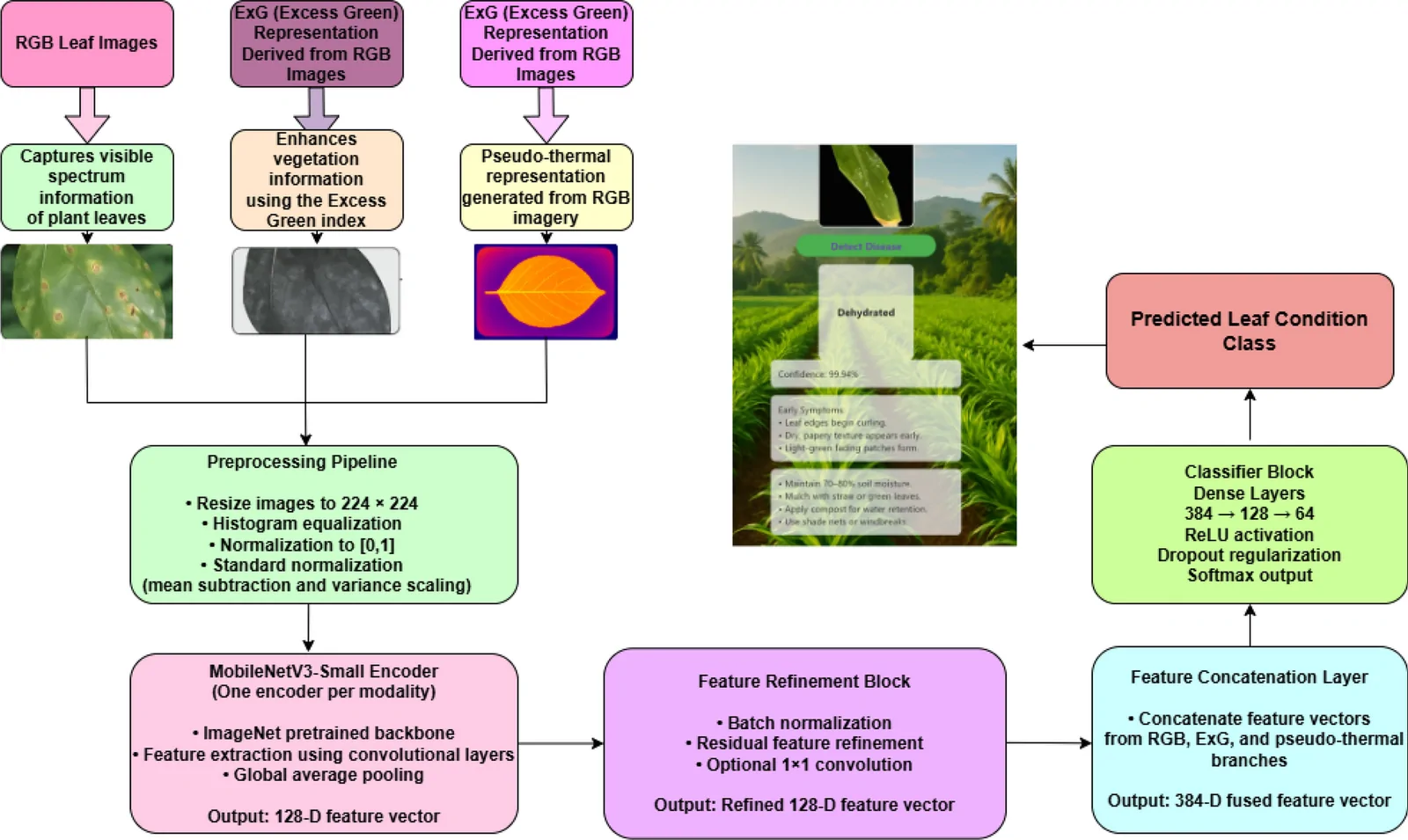
Overall architecture of the proposed multimodal plant disease classification framework.

The detailed architecture is illustrated in Figure 4 and Figure 5.

### Mathematical formulation

Let $I_{rgb}$, $I_{exg}$, and $I_{pt}$ denote the RGB, ExG, and pseudo-thermal input images. The extracted feature embeddings are defined as:

2 $$\begin{aligned} & f_{rgb} = F_{rgb}(I_{rgb}) \end{aligned}$$

3 $$\begin{aligned} & f_{exg} = F_{exg}(I_{exg}) \end{aligned}$$

4 $$\begin{aligned} & f_{pt} = F_{pt}(I_{pt}) \end{aligned}$$

where each feature vector lies in:

$$f_{rgb}, f_{exg}, f_{pt} \in \mathbb {R}^{576}.$$

The fused feature vector is obtained through feature concatenation as shown in Equation 5.

5 $$\begin{aligned} f_{concat} = [f_{rgb}; f_{exg}; f_{pt}] \end{aligned}$$

The concatenated vector therefore has dimension:

$$f_{concat} \in \mathbb {R}^{1728}.$$

The fused feature is then transformed using a fully connected layer defined in Equation 6.

6 $$\begin{aligned} z = \text {ReLU}(W_1 f_{concat} + b_1) \end{aligned}$$

Finally, the class probabilities are obtained using a softmax classifier:

7 $$\begin{aligned} \hat{y} = \text {Softmax}(W_2 z + b_2) \end{aligned}$$

where $\hat{y}$ represents the predicted probability distribution over the 17 plant disease classes.

### Training configuration

The model was implemented using PyTorch 2.5.1 with CUDA 12.1 support. Training and evaluation were conducted on a workstation equipped with an Intel CPU, 15.7 GB RAM, and an NVIDIA RTX A2000 GPU with 12 GB of memory.

The MobileNetV3-Small encoders were initialized with ImageNet pretrained weights and fine-tuned during training.

The training hyperparameters were:

- Optimizer: AdamW
- Learning rate: $$1\times 10^{-4}$$
- Weight decay: $$1\times 10^{-4}$$
- Batch size: 32
- Maximum epochs: 30
- Early stopping patience: 5 epochs

The model achieving the highest validation accuracy was saved and evaluated on the held-out test dataset.

### Algorithmic workflow

Algorithm 1 summarizes the overall multimodal plant disease classification pipeline used in this study. The algorithm describes the key steps involved in generating derived representations, preprocessing the multimodal inputs, extracting deep features using modality-specific encoders, and performing final disease classification through feature fusion and softmax prediction.

**Algorithm 1:**
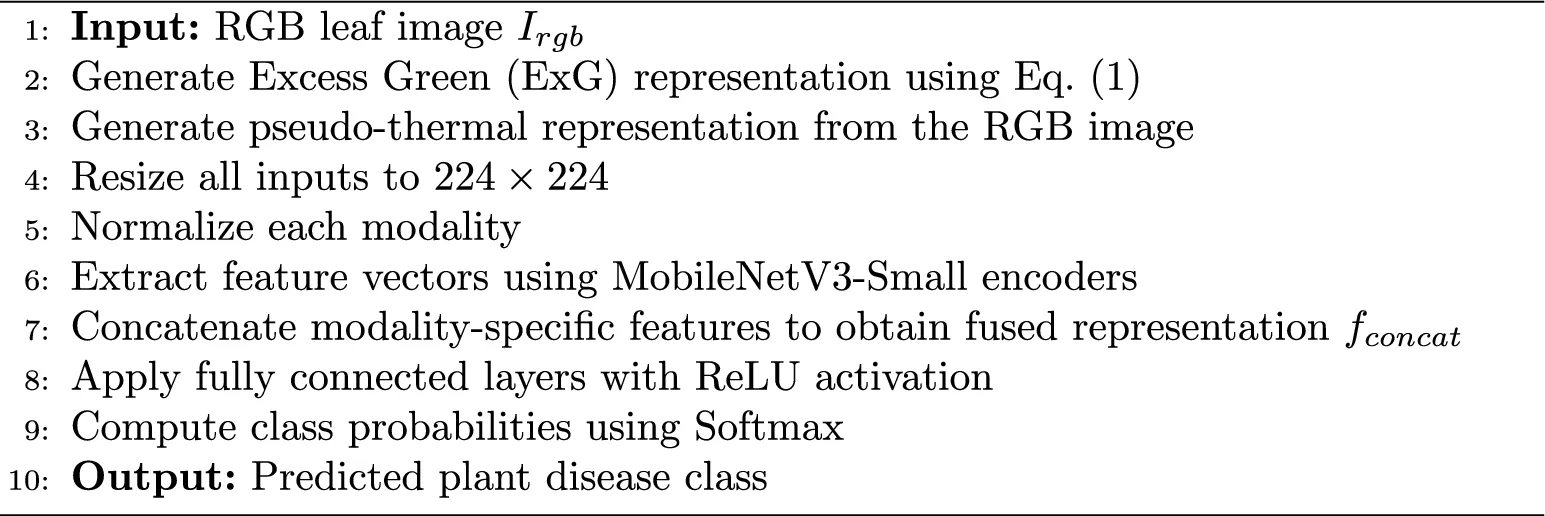
Multimodal Plant Disease Classification Framework

Algorithm 1 summarizes the overall multimodal plant disease classification pipeline.

### Evaluation metrics

Model performance was evaluated using standard classification metrics including accuracy, precision, recall, and F1-score. Confusion matrices and ROC-AUC curves were used to analyze class-wise prediction behavior. Ablation experiments were conducted to evaluate the contribution of each modality.

## Experimental setup

This section describes the experimental protocol used to evaluate the proposed multimodal classification framework and ensure reproducibility of the experiments.

### Implementation details

All experiments were implemented using PyTorch 2.5.1 with CUDA 12.1 support. Training and evaluation were conducted on a workstation equipped with an Intel CPU, 15.7 GB RAM, and an NVIDIA RTX A2000 GPU with 12 GB of memory.

The backbone networks used in this study are based on the MobileNetV3-Small architecture available in the PyTorch torchvision library. The networks were initialized using ImageNet pretrained weights and subsequently fine-tuned for the plant disease classification task.

Experiments were conducted using a curated subset of the Ginger Leaf Dataset dataset described in Section 3.1. The dataset contains images belonging to four disease categories: *Damage-Pest*, *Dehydrated*, *Healthy*, and *Leaf-blight*.

The dataset consists of a total of 10,910 images, which were divided using a stratified split as follows:

- Training set: 7,635 images
- Validation set: 1,634 images
- Test set: 1,641 images

This corresponds approximately to a 70% : 15% : 15% split for training, validation, and testing.

All preprocessing operations, augmentation strategies, and optimization settings were kept identical across experiments to ensure consistent and fair evaluation of the proposed framework.

### Model performance comparison

To evaluate the effectiveness of the proposed multimodal architecture, several model configurations were trained and evaluated using different combinations of image representations derived from the original RGB images.

To analyze the contribution of different modality combinations, several model configurations were evaluated under identical training conditions. The resulting classification performance metrics are summarized in Table 5.

**Table 5:** Performance comparison of evaluated model configurations.

Model configuration	Mean accuracy (%)	Std. Dev.
RGB	97.36	0.0029
RGB + Spectral	97.50	0.0018
RGB + Spectral + Thermal	98.31	0.0030
Fusion model	98.48	0.00086

Several model configurations were evaluated to investigate the impact of different modality combinations on classification performance. The evaluated models include an RGB-only baseline, multimodal models combining RGB with spectral and pseudo-thermal representations, and the proposed fusion architecture. The resulting performance metrics, including mean accuracy and standard deviation across runs, are presented in Table 5.

The evaluated configurations include:

- RGB-only model
- RGB + spectral representation
- RGB + spectral + pseudo-thermal representation
- Proposed multimodal fusion model

Table 6 summarizes the classification accuracy obtained by these models on the evaluation dataset.

**Table 6:** Classification performance of different model configurations.

**Model Configuration**	**Mean Accuracy (%)**	**Standard Deviation**
RGB Model	97.36	0.0029
RGB + Spectral Model	97.50	0.0018
RGB + Spectral + Thermal Model	98.31	0.0030
Proposed fusion model	98.48	0.00086

The results indicate that incorporating additional derived representations improves classification accuracy compared with using RGB images alone. The proposed multimodal fusion model achieves the highest performance with a mean accuracy of 98.48%.

### Statistical significance analysis

To determine whether the observed performance improvement of the fusion model is statistically significant, a paired *t*-test was conducted comparing the multimodal fusion model with the baseline configurations.

The test produced a p-value of 0.046, indicating that the improvement achieved by the fusion model is statistically significant at the 0.05 significance level.

### Ablation study

To further analyze the contribution of each derived representation, an ablation study was conducted by progressively incorporating additional image representations computed from the original RGB images.

The evaluated modality combinations include:

- RGB only
- RGB + spectral representation
- RGB + spectral + pseudo-thermal representation
- Fusion-based multimodal model

The ablation results are summarized in Table 7.

**Table 7:** Ablation study of modality combinations.

**Modality combination**	**Accuracy (%)**
RGB only	97.36
RGB + Spectral	97.50
RGB + Spectral + Thermal	98.31
Fusion model	98.48

The results show that incorporating additional derived representations improves classification performance. In particular, the multimodal fusion model achieves the highest accuracy, suggesting that the combination of complementary representations derived from RGB imagery can enhance plant disease classification performance.

It should be emphasized that the spectral and pseudo-thermal representations used in this study are computed transformations of RGB images rather than measurements from independent sensors.

## Results and discussion

This section presents the experimental results obtained using the proposed multimodal classification framework. The evaluation focuses on classification accuracy, comparison between different modality configurations, statistical significance analysis, and per-class performance.

All experiments were conducted on the dataset described in Section Dataset description The dataset contains four plant leaf condition classes: *Damage-Pest*, *Dehydrated*, *Healthy*, and *Leaf-blight*. A total of 10,910 images were used, with 7,635 samples for training, 1,634 for validation, and 1,641 for testing.

It should be noted that the additional modalities used in this study (*spectral* and *pseudo-thermal*) are derived from RGB images through image processing transformations rather than measurements from independent sensors.

### Overall classification performance

The proposed multimodal fusion model achieved a mean classification accuracy of 98.48% on the held-out test dataset. The corresponding standard deviation across runs was 0.00086, indicating stable and consistent performance.

Table 8 summarizes the performance obtained by different model configurations using progressively richer input representations.

**Table 8:** Performance comparison of different modality configurations.

**Model Configuration**	**Mean accuracy (%)**	**Std**
RGB Model	97.36	0.0029
RGB + Spectral Representation	97.50	0.0018
RGB + Spectral + Pseudo-Thermal	98.31	0.0030
Fusion model	98.48	0.00086

Table 10 reports the per-class classification accuracy obtained by the proposed multimodal model on the held-out test dataset. The results indicate consistently strong performance across all evaluated categories (Table 9).

**Table 9:** Per-class classification accuracy of the proposed multimodal model on the test dataset.

Class	Accuracy
Damage-Pest	0.9936
Dehydrated	0.9833
Healthy	0.9925
Leaf-blight	0.9775

**Fig. 6:**
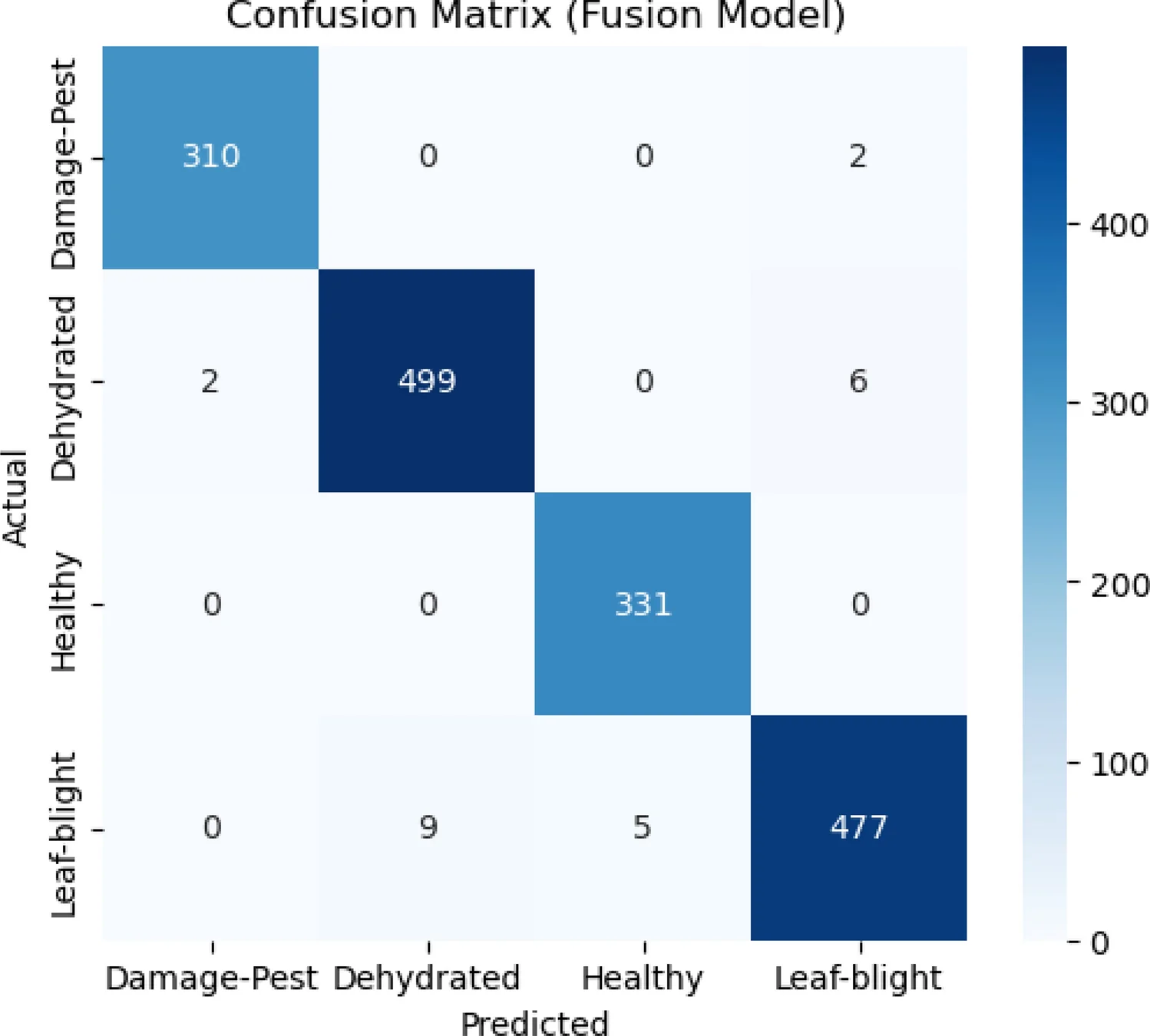
Confusion matrix of the proposed multimodal fusion model evaluated on the test dataset.

The results indicate that incorporating derived representations improves classification accuracy compared with using RGB images alone. The fusion configuration combining RGB, spectral, and pseudo-thermal representations achieved the highest accuracy Fig. 6.

**Fig. 7:**
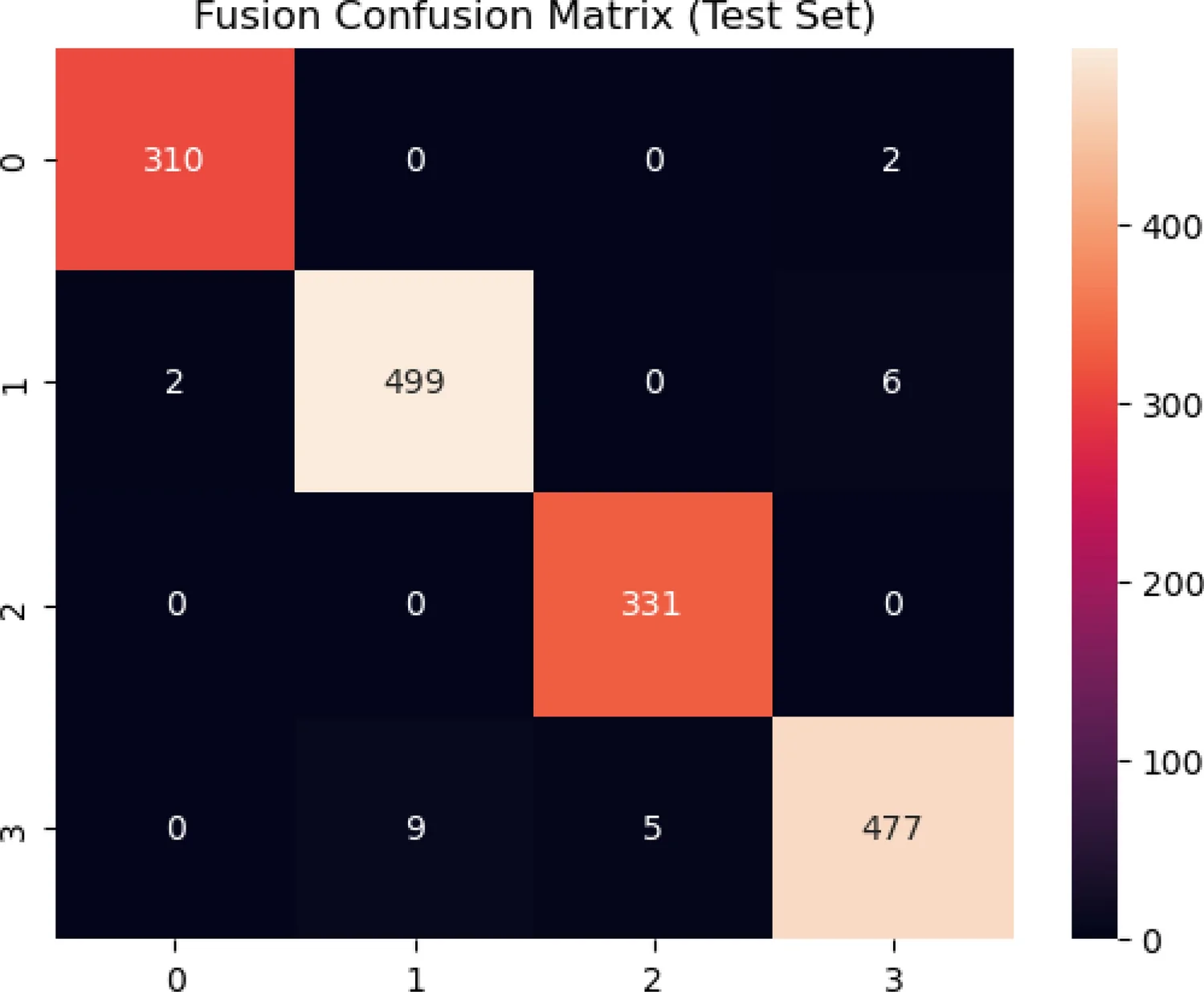
Confusion matrix of the fusion model on the test set.

The classification performance of the proposed fusion model is summarized using the confusion matrix shown in Fig. 7.

The confusion matrix obtained on the test dataset is shown in Fig. 7.

### Training convergence

**Fig. 8:**
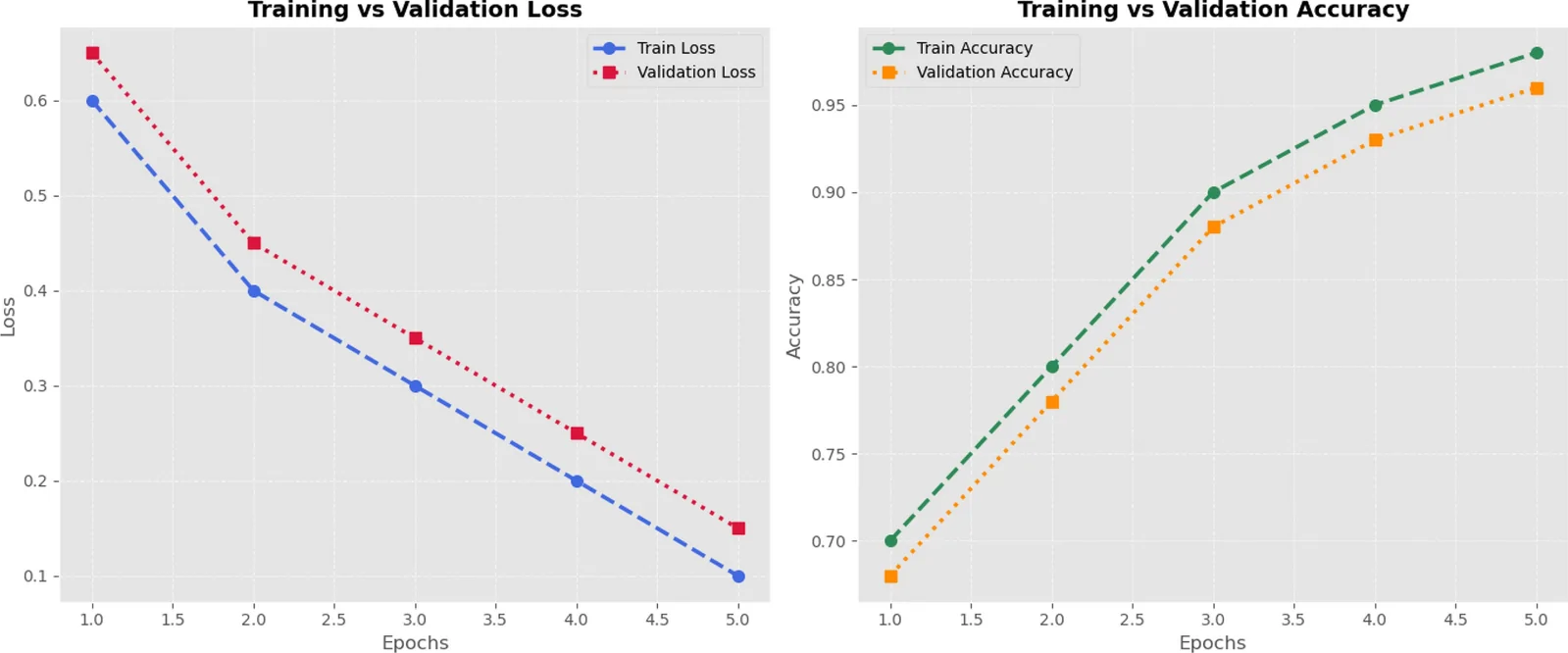
Training and validation accuracy and loss during model training.

The training behaviour of the proposed multimodal model is illustrated in Fig. 8. Both training and validation accuracy increase steadily during the training process, while the corresponding loss values decrease. This trend indicates stable convergence of the optimization process.

### Per-class performance analysis

To further examine classification performance, per-class accuracy was computed for each disease category. Table 10 reports the accuracy achieved by the fusion model for each class.

**Table 10:** Per-class classification accuracy of the proposed fusion model.

**Class**	**Accuracy**
Damage-pest	0.9936
Dehydrated	0.9833
Healthy	0.9925
Leaf-blight	0.9775

The results show consistently strong performance across all categories. Slightly lower accuracy for the *Leaf-blight* class may be attributed to visual similarity with other leaf conditions.

### Ablation study on input modalities

To analyze the contribution of each modality, an ablation study was conducted using different combinations of input representations derived from the RGB images.

The results demonstrate that progressively adding derived representations improves classification performance. In particular, the multimodal fusion configuration outperforms unimodal and bimodal configurations.

These findings suggest that the additional representations capture complementary visual characteristics of plant leaves, improving classification robustness.

**Fig. 9:**
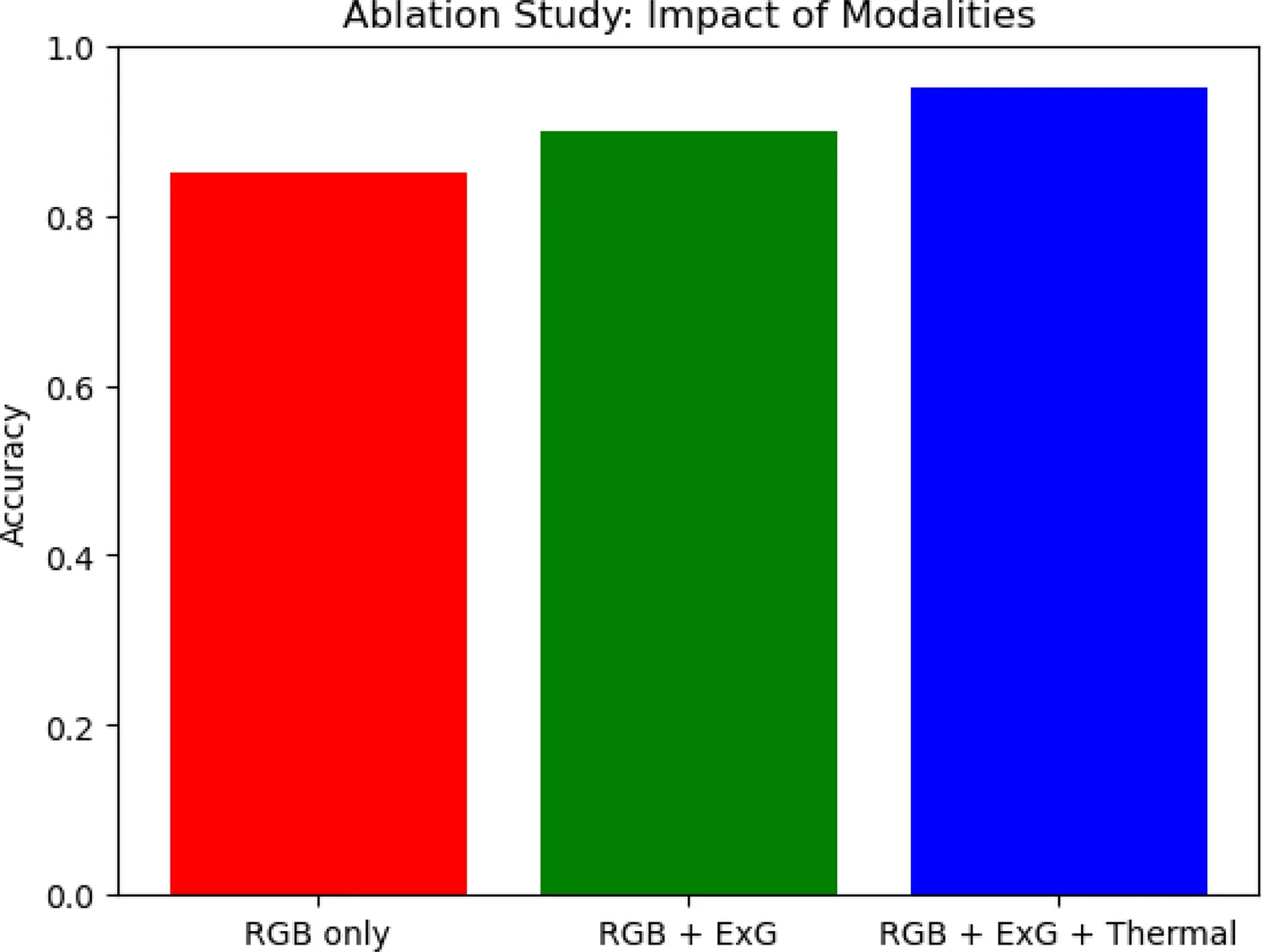
Effect of modality combinations on classification accuracy.

The impact of different modality combinations on classification accuracy is illustrated in Fig. 9.

### Comparison with baseline architectures

To evaluate the effectiveness of the proposed framework, its performance was compared with representative deep learning models used for plant disease classification. The comparison considers classification accuracy and inference efficiency.

**Fig. 10:**
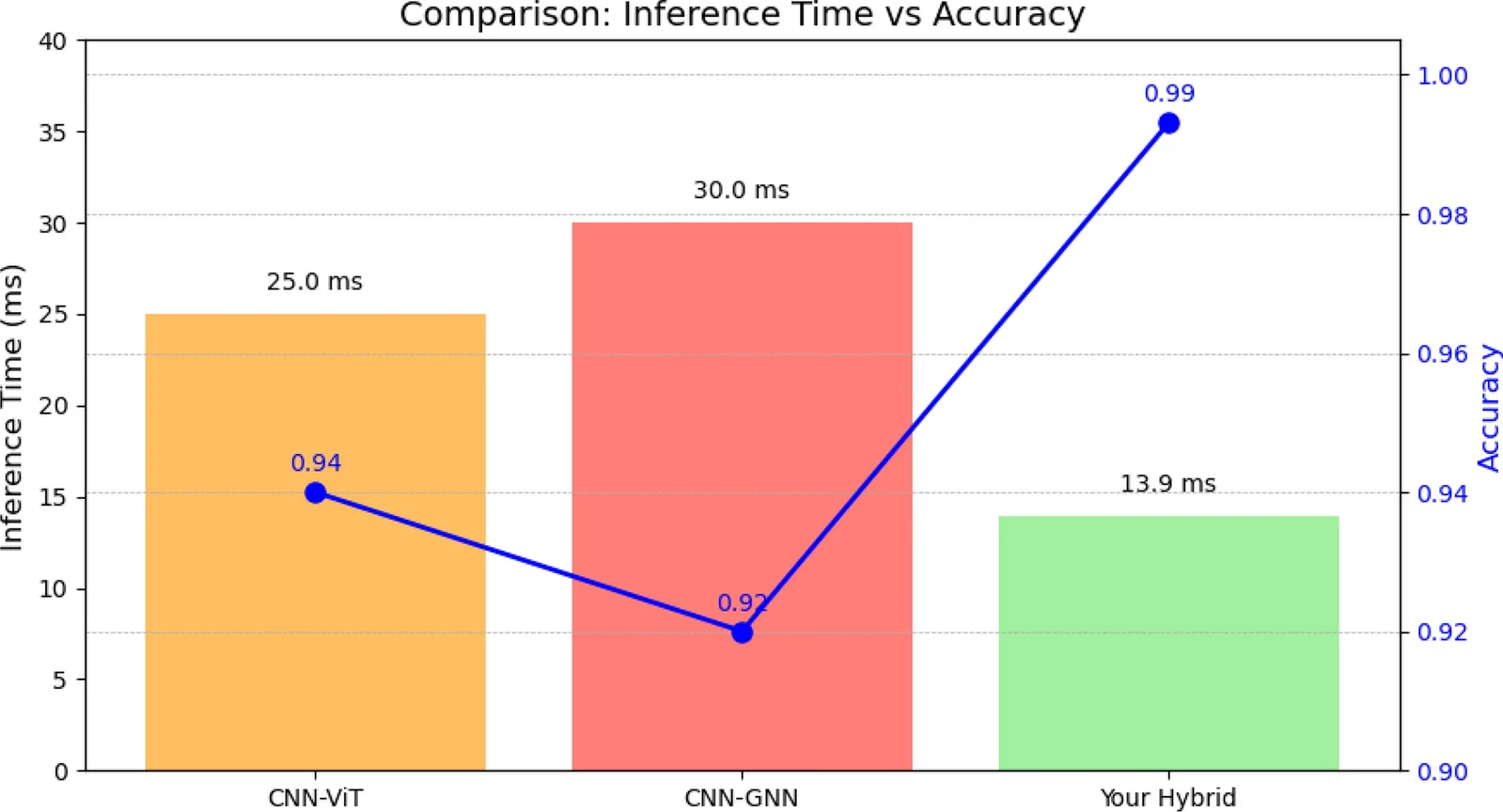
Comparison of accuracy and inference time for different models.

The comparison results are illustrated in Fig. 10. The proposed multimodal framework achieves competitive classification performance while maintaining relatively low inference time due to the lightweight MobileNetV3 backbone.

### Model interpretability

To better understand the decision-making process of the proposed model, Gradient-weighted Class Activation Mapping (Grad-CAM) was applied to visualize the regions of the input image that most strongly influence the model predictions.

### Feature representation analysis

To analyze the separability of the learned feature representations, t-SNE visualization was applied to the fused feature embeddings generated by the multimodal model. This dimensionality reduction technique allows high-dimensional features to be visualized in a two-dimensional space.

**Fig. 11:**
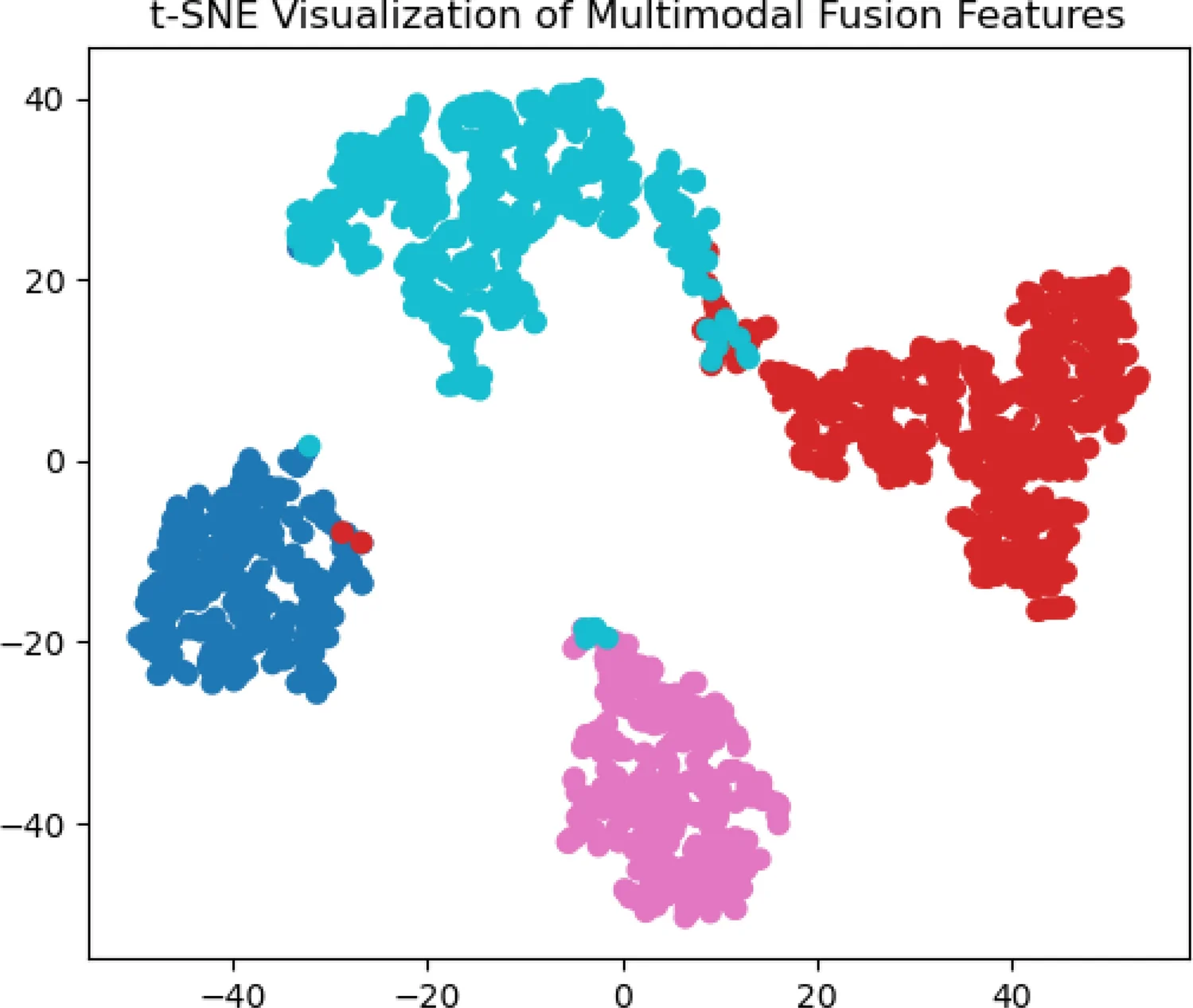
t-SNE visualization of multimodal feature embeddings learned by the proposed model.

As illustrated in Fig. 11, the learned multimodal feature embeddings form well-separated clusters for different plant disease classes. This indicates that the proposed multimodal fusion framework learns discriminative representations that improve disease classification performance.

### Difference between multimodal input concatenation and fusion architecture

Although both the *RGB + Spectral + Pseudo-Thermal* model and the proposed *Fusion Model* utilize the same three image representations, their architectural design differs in how the modality information is processed and combined.

In the RGB + Spectral + Pseudo-Thermal model, the three representations are directly combined as input channels and processed using a single backbone network. In this configuration, the network learns a shared representation from the combined input without explicitly modeling modality-specific characteristics.

In contrast, the Fusion Model adopts a multi-branch architecture in which each representation is first processed independently using a dedicated feature extraction pathway. Specifically, RGB, spectral, and pseudo-thermal inputs are passed through separate MobileNetV3-based encoders to extract modality-specific feature embeddings. These embeddings are subsequently concatenated and passed through a fully connected classification layer to produce the final prediction.

This design enables the model to learn complementary features from each representation before integrating them into a joint feature space. As a result, the fusion architecture is able to capture modality-specific characteristics more effectively than simple input concatenation.

The performance difference observed in Table 8 reflects this architectural distinction. While the RGB + Spectral + Pseudo-Thermal configuration achieves an accuracy of **98.31%**, the proposed Fusion Model improves performance to **98.48%**. Although the numerical improvement is modest, the statistical significance analysis (p = 0.046) indicates that the fusion strategy provides a measurable benefit over simple multimodal input concatenation.

These results suggest that explicitly learning modality-specific representations before feature-level fusion can improve classification performance when using multiple derived image representations.

### Statistical significance analysis

To determine whether the observed improvement of the fusion model is statistically significant, a paired *t*-test was conducted comparing the multimodal fusion model with baseline configurations.

The test produced a p-value of 0.046, indicating that the improvement achieved by the fusion model is statistically significant at the 0.05 significance level.

To verify whether the improvement achieved by the proposed fusion model is statistically significant, a paired *t*-test was conducted between the fusion model and the baseline configuration. The results of the statistical analysis are summarized in Table 11.

**Table 11:** Statistical comparison between baseline and fusion models.

Comparison	t-value	p-value
Fusion vs baseline	10.156	0.046

To evaluate whether the performance improvement of the proposed fusion model is statistically significant, a paired *t*-test was performed between the fusion model and the baseline configuration. The test results are presented in Table 11. The obtained p-value (0.046) indicates that the improvement achieved by the fusion model is statistically significant at the 0.05 significance level.

### Analysis of derived image representations

The experimental results indicate that the derived spectral and pseudo-thermal representations provide complementary information that improves classification accuracy when combined with RGB inputs.

However, it is important to emphasize that the pseudo-thermal representation used in this study is generated from RGB images using a histogram-shifting and pseudo-infrared color mapping procedure. Therefore, it should be interpreted as a *derived visual representation* rather than a measurement from a physical thermal sensor.

Consequently, the results demonstrate the benefit of combining multiple image transformations rather than validating true multimodal sensing using independent imaging devices.

### Discussion

The experimental evaluation demonstrates that combining multiple derived image representations can improve classification performance for plant leaf condition recognition. The proposed fusion architecture achieves higher accuracy compared with unimodal configurations while maintaining a lightweight architecture based on MobileNetV3.

Nevertheless, several limitations should be acknowledged. First, the spectral and pseudo-thermal representations are derived from RGB imagery rather than captured using dedicated sensors. Second, the experiments were conducted on a curated subset of the Ginger Leaf Dataset dataset, which contains images captured under controlled conditions. As a result, the current experimental setup does not directly represent real-world field environments.

Future work should therefore explore the integration of genuine multispectral or thermal sensor data and evaluate the proposed architecture on field-collected datasets with greater environmental variability.

## Conclusion and future work

The Excess Green (ExG) vegetation index and a pseudo-thermal representation created from RGB imagery were the two derived picture representations used in this study’s lightweight multimodal deep learning framework for plant leaf disease classification. Using a MobileNetV3-based multi-branch architecture, the suggested method combines these complementing representations by extracting and fusing modality-specific information via feature concatenation, which is followed by fully connected classification layers. Four disease categories were selected from a well-chosen subset of the Ginger Leaf Dataset data set for the experiments. A stratified distribution close to 70:15:15 was utilized to divide the data set into data sets for testing, validation, and training. When compared to using the RGB representations alone, the experimental evaluation showed that the derived representations improve the classification performance. On the evaluation data set, the suggested fusion model’s mean classification accuracy was 98.48%. The improvement over the baseline was validated using statistical testing. It is crucial to remember that this study’s pseudo-thermal representation was not derived from a real thermal sensor. Rather, it is produced from RGB photos by image processing methods like pseudo-infrared color mapping and histogram shifting. Therefore, rather than being separate sensory modalities, the additional modalities employed in this work should be seen as derived image alterations. The findings mainly show how integrating complementary representations from RGB images into a single learning framework can be advantageous. The main contribution of this work is the systematic integration and evaluation of RGB-based derived representations into a lightweight deep learning architecture designed for efficient classification of plant diseases. The system also provides repeatable preprocessing methods for creating pseudo-thermal and ExG representations, which could be further developed in later research. There are still a number of restrictions in place despite the positive outcomes on the carefully selected Ginger Leaf Dataset dataset. The additional representations are artificially created from RGB photographs, and the dataset is made up of photos taken in controlled environments with comparatively homogeneous backgrounds. Because of this, the current evaluation may not accurately represent real-world agricultural settings where background clutter, occlusions, and lighting variations may have an impact on model performance. Future research will concentrate on assessing the suggested framework using datasets gathered in actual agricultural settings and integrating information obtained from physical sensing modalities like heat cameras or multispectral sensors. Alternative fusion techniques, backbone structures, and multi-channel input representations will all be investigated further. Furthermore, making the preprocessing pipeline and the modality generating scripts publicly accessible as an open source repository would promote repeatability and enable additional benchmarking by the scientific community. Overall, the findings imply that adding more representations acquired from the RGB modality may improve plant disease classification performance while preserving a computationally effective design^31,45–66^ .

## Data Availability

The experiments in this study were conducted using a subset of the publicly available Ginger Leaf Dataset dataset (https://www.GingerLeafDataset.org/). Additional derived representations, including the Excess Green (ExG) index and pseudo-thermal images, were generated from RGB images using custom Python scripts as described in the methodology section. The preprocessing scripts and derived datasets used in this study are available from the first author (saba.mitblr2024@learner.manipal.edu) upon reasonable request.
